# Effects of myocardial sheetlet sliding on left ventricular function

**DOI:** 10.1007/s10237-023-01721-6

**Published:** 2023-05-06

**Authors:** Yu Zheng, Wei Xuan Chan, Sonia Nielles-Vallespin, Andrew D. Scott, Pedro F. Ferreira, Hwa Liang Leo, Choon Hwai Yap

**Affiliations:** 1 Department of Biomedical Engineering, National University of Singapore, Singapore, Singapore; 2 Department of Bioengineering, Imperial College London, London, UK; 3 British Heart Foundation Centre of Research Excellence, Imperial College London, London, UK; 4 Cardiovascular Magnetic Resonance Unit, Royal Brompton Hospital, London, UK; 5 National Heart and Lung Institute, Imperial College London, London, UK

**Keywords:** Myocardial sheetlet sliding, Sheetlet orientation, Shear stiffness, LV function, Finite element, LV hypertrophy, LV dilation

## Abstract

Left ventricle myocardium has a complex micro-architecture, which was revealed to consist of myocyte bundles arranged in a series of laminar sheetlets. Recent imaging studies demonstrated that these sheetlets re-orientated and likely slided over each other during the deformations between systole and diastole, and that sheetlet dynamics were altered during cardiomyopathy. However, the biomechanical effect of sheetlet sliding is not well-understood, which is the focus here. We conducted finite element simulations of the left ventricle (LV) coupled with a windkessel lumped parameter model to study sheetlet sliding, based on cardiac MRI of a healthy human subject, and modifications to account for hypertrophic and dilated geometric changes during cardiomyopathy remodeling. We modeled sheetlet sliding as a reduced shear stiffness in the sheet-normal direction and observed that (1) the diastolic sheetlet orientations must depart from alignment with the LV wall plane in order for sheetlet sliding to have an effect on cardiac function, that (2) sheetlet sliding modestly aided cardiac function of the healthy and dilated hearts, in terms of ejection fraction, stroke volume, and systolic pressure generation, but its effects were amplified during hypertrophic cardiomyopathy and diminished during dilated cardiomyopathy due to both sheetlet angle configuration and geometry, and that (3) where sheetlet sliding aided cardiac function, it increased tissue stresses, particularly in the myofibre direction. We speculate that sheetlet sliding is a tissue architectural adaptation to allow easier deformations of the LV walls so that LV wall stiffness will not hinder function, and to provide a balance between function and tissue stresses. A limitation here is that sheetlet sliding is modeled as a simple reduction in shear stiffness, without consideration of micro-scale sheetlet mechanics and dynamics.

**Supplementary Information:**

The online version contains supplementary material available at 10.1007/s10237-023-01721-6.

## Introduction

The left ventricular (LV) myocardium has a complex and well-organized microstructure. Investigation of LV tissue revealed that it has transmurally (epicardial-to-endocardial) varying myocyte orientation with an average angle close to 0° (LeGrice et al. 1995; Streeter and Bassett 1966; Streeter et al. 1969). On top on this, myocytes have transverse angles, where they depart from the LV wall plane and have a component of their orientation toward the endocardium (Teh et al. 2017). This transverse angle is proposed to have a transmural and basal–apical variation (Vendelin et al. 2002).

**Fig. 1:**
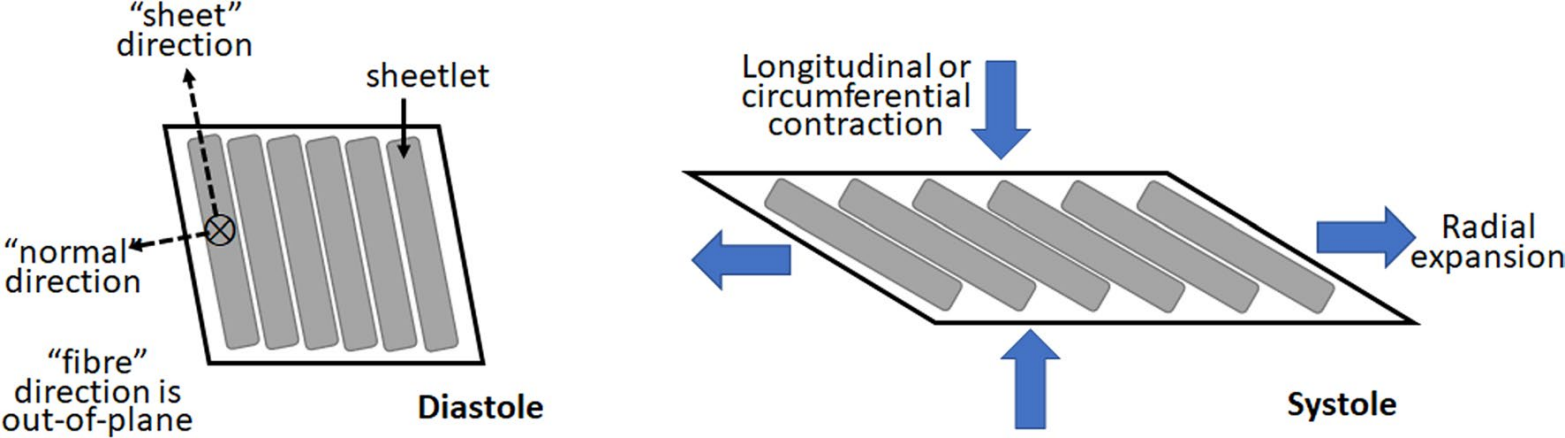
Schematic of the hypothesis on how sheetlet sliding can aid diastole-to-systole myocardial deformations. Sheetlets are composed of groups of myocytes connected in a sheet-like structure. The sliding of sheetlets over each other has been hypothesized to enable easier shear deformation in the sheet-normal direction, enabling easier contractile deformations in the longitudinal and circumferential directions and expansion deformation in the radial direction during systole

Recent histology studies revealed that the LV myocytes are organized into laminar microstructures, which were given the term sheetlets, and were about three to six myocytes in thickness (LeGrice et al. 1995; Wilson et al. 2022). Diffusion tensor cardiac magnetic resonance imaging (DTCMR) further revealed that these sheetlets have specific systolic–diastolic dynamics, where the sheetlet angle or orientation experiences a cyclic tilt away from the LV wall plane during systolic contraction (Ferreira et al. 2014; Nielles-Vallespin et al. 2017). It was proposed that sheetlets slide over each other during contractions to enable easier cardiac deformations (Harrington et al. 2005), where the sliding and tilting of the sheetlet naturally leads to longitudinal or circumferential shortening, and radial lengthening of the myocardium (Fig. 1). Studies further demonstrated that baseline sheetlet orientations as well as sheetlet orientation dynamics were altered during dilated and hypertrophic cardiomyopathy (DCM and HCM) (Nielles-Vallespin et al. 2017).

Biomechanical testing of human and porcine myocardium has corroborated these findings on sheetlet sliding (Dokos et al. 2002; Sommer et al. 2015). Sommer et al. performed shear mechanical testing in all 6 directions and found that the myocardium had especially low shear stiffness in the sheet-normal, normal-sheet, sheet-fiber, and fiber-sheet shear directions (direction conventions indicated in Fig. 3). These reduced stiffnesses were likely enabled by the sheetlet sliding mechanism (Sommer et al. 2015).

However, to date, the biomechanical effects of sheetlet sliding have not been studied in detail and are not well understood. Here, we conduct finite element (FE) simulations of the LV myocardium, to provide additional evidence for sheetlet sliding, and using reduced shear stiffness to model sheetlet sliding, investigate its effects on cardiac function and tissue stresses in healthy, HCM and DCM LV geometries. Finite element method (FEM) has in the past been very successful at revealing biomechanics details in health and disease (Ong et al. 2021; Shavik et al. 2017) and is thus a very suitable tool for this investigation.

## Methods

### Left ventricle geometry and shape morphing

**Fig. 2:**
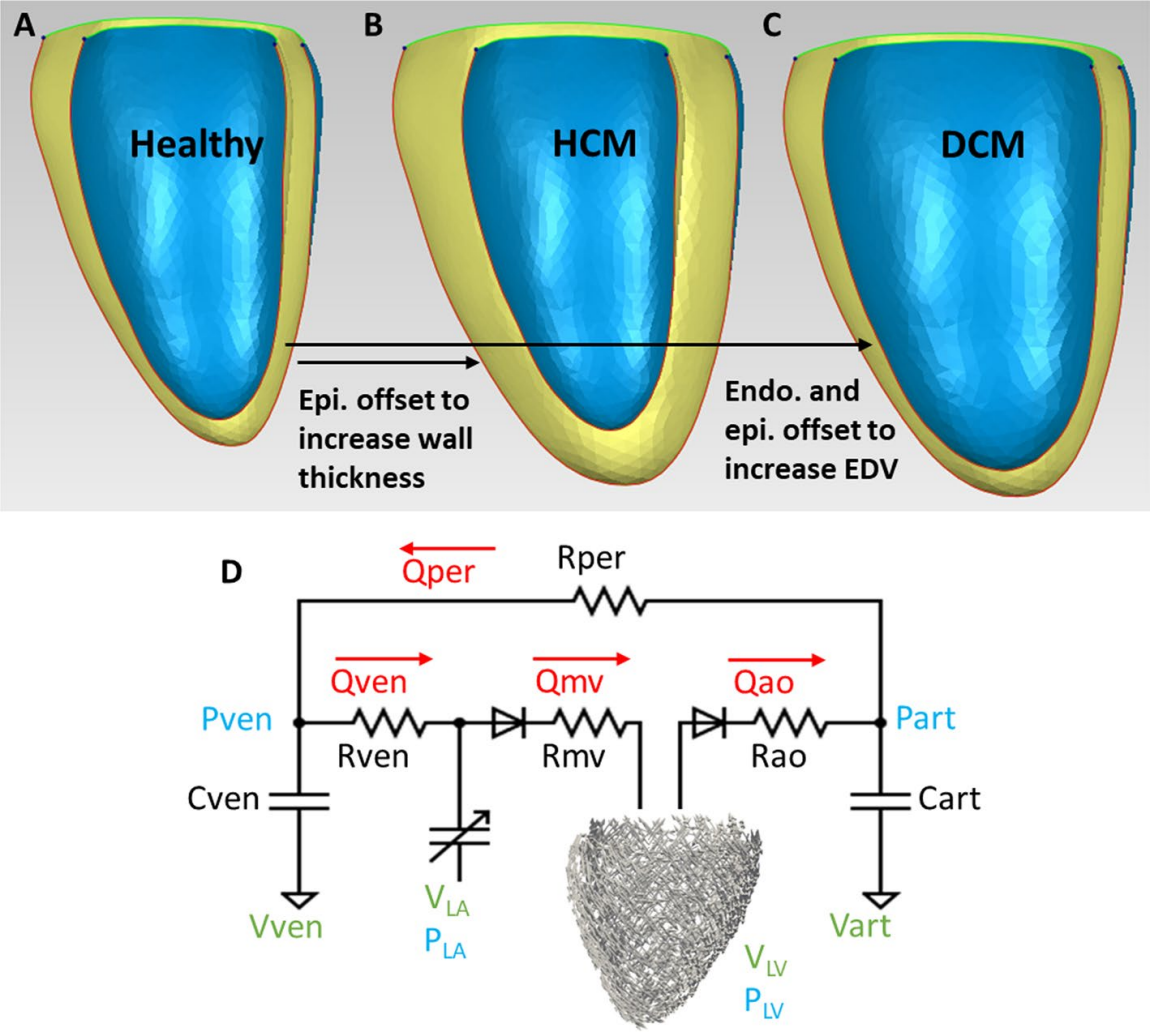
End-diastolic LV geometries used in our FEM simulations. **a** Healthy LV myocardium reconstructed from CMR images. **b** LV myocardium with wall hypertrophy to represent HCM geometry, obtained by offsetting the epicardium surface of the healthy LV to double wall thickness while retaining the end-diastolic volume. **c** LV myocardium with dilation, obtained by offset both the endocardium and epicardium surfaces of the healthy LV to increase the end-diastolic volume by 1.8 times while keeping wall thicknesses constant. **d** The closed loop windkessel lumped-parameter circulatory model that was coupled with the LV FEM simulation. *C—*compliance, *R—*resistance, *P—*pressure, *V—*volume, *Q—*volumetric flow rate. subscripts *art*—arterial, *ven—*venous, *per—*peripheral vascular, *ao—*aortic valve, *mv—*mitral valve, *LA—*left atrium, *LV—*left ventricle

A 3D left ventricular myocardium at end-diastole (ED) was reconstructed from the cardiac magnetic resonance (CMR) imaging of a healthy adult human volunteer. The protocol was approved by the Surrey Research Ethics Committee (protocol 10/H0701/112), and informed consent was obtained from all participants. Segmentation and reconstruction were performed with VMTK (www.vmtk.org), while smoothing was performed with Geomagic Studio (Geomagic Inc., Morrisville, NC, USA). The healthy LV model was later morphed to one with concentric hypertrophy and one with dilated eccentric hypertrophy, to investigate biomechanics during such LV geometry alternations. Concentric hypertrophy was modeled as a 100% increase in wall thickness on the load-free geometry, via an offset of the epicardium outwards, while dilation was modeled as an 80% increase in end-diastolic volume (EDV) without a change in wall thickness on the load-free geometry, via an offset of both the epicardium and endocardium outwards (Fig. 2). These were in accordance with clinical measurements of wall thicknesses and EDV for HCM and DCM patients (Nielles-Vallespin et al. 2017).

LV geometries were meshed with 1744–1925 tetrahedron elements, with an average element cell volume of 0.07–0.13 ml. Mesh convergence test was as previous performed in (Ong et al. 2021). To calculate the load-free geometry, we assumed that the end diastolic pressure (EDP) of the healthy LV to be 5 mmHg (Westermann et al. 2008), and we estimated the unloading deformation from the end-diastole state to the load-free state as the inverse of the loading deformation for the same pressure difference. Once the load-free geometry was obtained, we morphed the healthy load-free LV to HCM and DCM LV as described above.

### Computational finite element modeling framework

A computational finite element method was employed from the previous studies coupled with an LV close-loop windkessel circulatory (Ong et al. 2021; Shavik et al. 2018). Simulations were conducted with FeniCS (www.fenicsproject.org), by minimizing the Lagrangian function detailed by Shavik et al. (2018). FEM utilized a transversely isotropic Fung-type strain energy function to back compute the loading-free geometry with assigned end-diastolic pressure and myocardial stiffness information. The passive myocardial constitutive model involving a Fung-type strain energy function, ***W*** (Guccione et al. 1991), which was utilized by the previous simulation work with good results (Shavik et al. 2018), was given by:

1a $$\begin{array}{*{20}c} {{\varvec{W}} = \frac{1}{2}C(e^{Q} - 1}) \\ \end{array}$$

where *Q* was calculated as:

1b $$\begin{array}{c}Q={B}_{{\varvec{f}}{\varvec{f}}}{{\varvec{E}}}_{{\varvec{f}}{\varvec{f}}}^{2}+{B}_{{\varvec{s}}{\varvec{s}}}{{\varvec{E}}}_{{\varvec{s}}{\varvec{s}}}^{2}+{B}_{{\varvec{n}}{\varvec{n}}}{{\varvec{E}}}_{{\varvec{n}}{\varvec{n}}}^{2}+{B}_{{\varvec{n}}{\varvec{s}}}\left({{\varvec{E}}}_{{\varvec{s}}{\varvec{n}}}^{2}+{{\varvec{E}}}_{{\varvec{n}}{\varvec{s}}}^{2}\right)+ {B}_{{\varvec{f}}{\varvec{s}}}\left({{\varvec{E}}}_{{\varvec{f}}{\varvec{s}}}^{2}+{{\varvec{E}}}_{{\varvec{s}}{\varvec{f}}}^{2})+{B}_{{\varvec{f}}{\varvec{n}}}({{\varvec{E}}}_{{\varvec{f}}{\varvec{n}}}^{2}+{{\varvec{E}}}_{{\varvec{n}}{\varvec{f}}}^{2}\right)\end{array}$$

*C* in the Eq. (1a) was the global myocardial stiffness, while ${B}_{{\varvec{f}}{\varvec{f}}}$, ${B}_{{\varvec{s}}{\varvec{s}}}$, ${B}_{{\varvec{n}}{\varvec{n}}}$, ${B}_{{\varvec{n}}{\varvec{s}}}$, and ${B}_{{\varvec{f}}{\varvec{s}}}$ and ${B}_{{\varvec{f}}{\varvec{n}}}$ were the passive material parameters in various directions, where ***f***, ***s***, and ***n*** denoting the myocardial fiber, sheetlet, and normal directions, respectively. As shown in Fig. 1, ***f*** is the orientation of the myocyte, ***s*** is perpendicular to ***f*** in the sheetlet plane, while ***n*** is normal to the sheetlet plane. In particular, ${B}_{{\varvec{n}}{\varvec{s}}}$ denoted the shear stiffness in the sheet and normal directions, and was the parameter that was reduced to model sheetlet sliding. ***E*** was the Green–Lagrange strain tensor. The incompressible criteria were enforced in the FE solver, by minimizing the deformation Jacobian (Shavik et al. 2018). For simulations of healthy LV, C was assumed to be 100 Pa, which was consistent with past simulation work (Rumindo et al. 2020; Shavik et al. 2018), but for HCM and DCM diseased conditions, they could increase to 300 Pa and 200 Pa, respectively, as informed by findings that diastolic dysfunction increases myocardial stiffness by 2–3 times (Klotz et al. 2005; Wang et al. 2018; Westermann et al. 2008).

The Guccione model was employed to simulate the active myocardial contractile mechanical behavior (Shavik et al. 2018), which is modeled as the maximum tension (${T}_{max}$) multiplied by a calcium activation curve over time, details of which are given in the Appendix. For healthy LVs, ${T}_{\mathrm{max}}$ was assumed to be 150 kPa, but for diseased HCM and DCM LVs, they were reduced to be 75 or 105 kPa, respectively, in accordance with previous work by Shavik et al. supporting reduced active tension in HCM (Shavik et al. 2021) and by Meurs et al. (2019) showing reduced active tension in DCM. The end-diastolic pressure (EDP) was assumed to be 7 mmHg for cardiomyopathy cases but 5 mmHg for healthy cases.

The boundary conditions for the FEM simulations were a constraint at the LV base on out-of-plane motion and a low-stiffness (60 Pa) spring constraint on the entire epicardial surface to emulate interactions with surrounding tissues to prevent the model from drifting away (Shavik et al. 2018). The direction and magnitude of the spring force were linear functions of the displacement vector of each epicardial point.

A simplified windkessel lumped-parameter model was coupled to the LV FEM, as shown in Fig. 2D. It consists of peripheral vascular and venous resistances (*R*_per_ and *R*_ven_) and aortic valve and mitral valve resistances (*R*_ao_ and *R*_mv_), and arterial and venous compliances (*C*_art_, and *C*_ven_). Initial volumes of arterial and venous were tuned together with values of resistances and compliances to obtain the expected pressure–volume loop for the normal LV. Thereafter, the same lumped parameter model was used for other LV geometries and cardiac contractilities and passive stiffnesses. Details of the lumped parameter model parameters are given in the *Appendix*. FEM simulations were conducted for 10 cycles to allow the lumped parameter model to converge.

### Estimation of myocardial normal stiffness and shear stiffness

To obtain the specification of the passive stiffness model, we performed numerical modeling of simple shear mechanical testing of a cuboid piece of myocardium using our passive stiffness model, to match data obtained by mechanical testing experiments (Dokos et al. 2002; Sommer et al. 2015). The relationship between myocardial passive stress (${{\varvec{\sigma}}}_{{\varvec{p}}}$_)_ and Green–Lagrange strain (${\varvec{E}}$) was modeled via finite strain theory as:

2 $$\begin{array}{*{20}c} {{\varvec{\sigma}}_{{\varvec{p}}} = \frac{1}{J}\varvec{F}\frac{{\partial {\varvec{W}}}}{{\partial {\varvec{E}}}}{\varvec{F}}^{T} } \\ \end{array}$$

where ${\varvec{F}}$ is the deformation gradient tensor and *J* is the Jacobian of the deformation gradient tensor ${\varvec{F}}$*.* According to Sommer et al., the myocardial stiffness in the fiber direction was about twice as stiff as in the cross-fiber direction from the biaxial extension testing (Sommer et al. 2015). Therefore, we specified ${B}_{{\varvec{f}}{\varvec{f}}}$ to be 29.8, twice that of ${B}_{{\varvec{s}}{\varvec{s}}}$ and ${B}_{{\varvec{n}}{\varvec{n}}}$, which were 14.9, in accordance with the previous FEM studies (Rumindo et al. 2020; Shavik et al. 2018).

To model the sheetlet sliding, we reduced ${B}_{{\varvec{s}}{\varvec{n}}}$ and ${B}_{{\varvec{n}}{\varvec{s}}}$ to be the same and reduced from other shear stiffness components ${B}_{{\varvec{f}}{\varvec{s}}}$, ${B}_{{\varvec{s}}{\varvec{f}}}$, ${B}_{{\varvec{n}}{\varvec{f}}}$ and ${B}_{{\varvec{f}}{\varvec{n}}}$. We further assumed ${B}_{{\varvec{f}}{\varvec{s}}}$, ${B}_{{\varvec{s}}{\varvec{f}}}$, ${B}_{{\varvec{n}}{\varvec{f}}}$ and ${B}_{{\varvec{f}}{\varvec{n}}}$ to be same. From Sommer et al. (2015), the shear stress to achieve a shear deformation of 0.4 in the ***f***-***s*** direction was 4.6 $\pm$ 1.0 kPa. From our modeling of simple shear in the ***f***-***s***, a ${B}_{{\varvec{f}}{\varvec{s}}}$ of 19.2 would match this behavior. From Sommer et al., the shear stress to achieve a shear deformation of 0.4 in the ***n***-***s*** direction was 2.2 $\pm$ 0.8 kPa. From our modeling of simple shear in the ***n***-***s*** direction, a ${B}_{{\varvec{n}}{\varvec{s}}}$ of 15.4 would match this behavior. We performed FEM modeling with four values of ${B}_{{\varvec{n}}{\varvec{s}}}$, 9.3 12.1, 15.0 and 17.8, representing -1.6, -1.0, -0.2, + 1.0 standard deviations from the mean ***n***-***s*** stress value from Sommer et al.’s data. This range of stiffness was within range investigated by previous FEM studies (Rumindo et al. 2020; Zhang et al. 2021).

We further tested reducing shear stiffness ${B}_{{\varvec{f}}{\varvec{s}}}$ and ${B}_{{\varvec{f}}{\varvec{n}}}$, on top of reducing ${B}_{{\varvec{n}}{\varvec{s}}}$, but found that ${B}_{{\varvec{f}}{\varvec{s}}}$ and ${B}_{{\varvec{f}}{\varvec{n}}}$ had very minimal influence on cardiac functions (Appendix Table 6), likely because cardiac deformations did not engage shear in these directions much. ${B}_{{\varvec{f}}{\varvec{s}}}$ and ${B}_{{\varvec{f}}{\varvec{n}}}$ were thus held constant value 19.2 in our simulations, and only reduced stiffness in ${B}_{{\varvec{n}}{\varvec{s}}}$ was used to model sheetlet sliding.

### Assignments of myocardial orientations

**Fig. 3:**
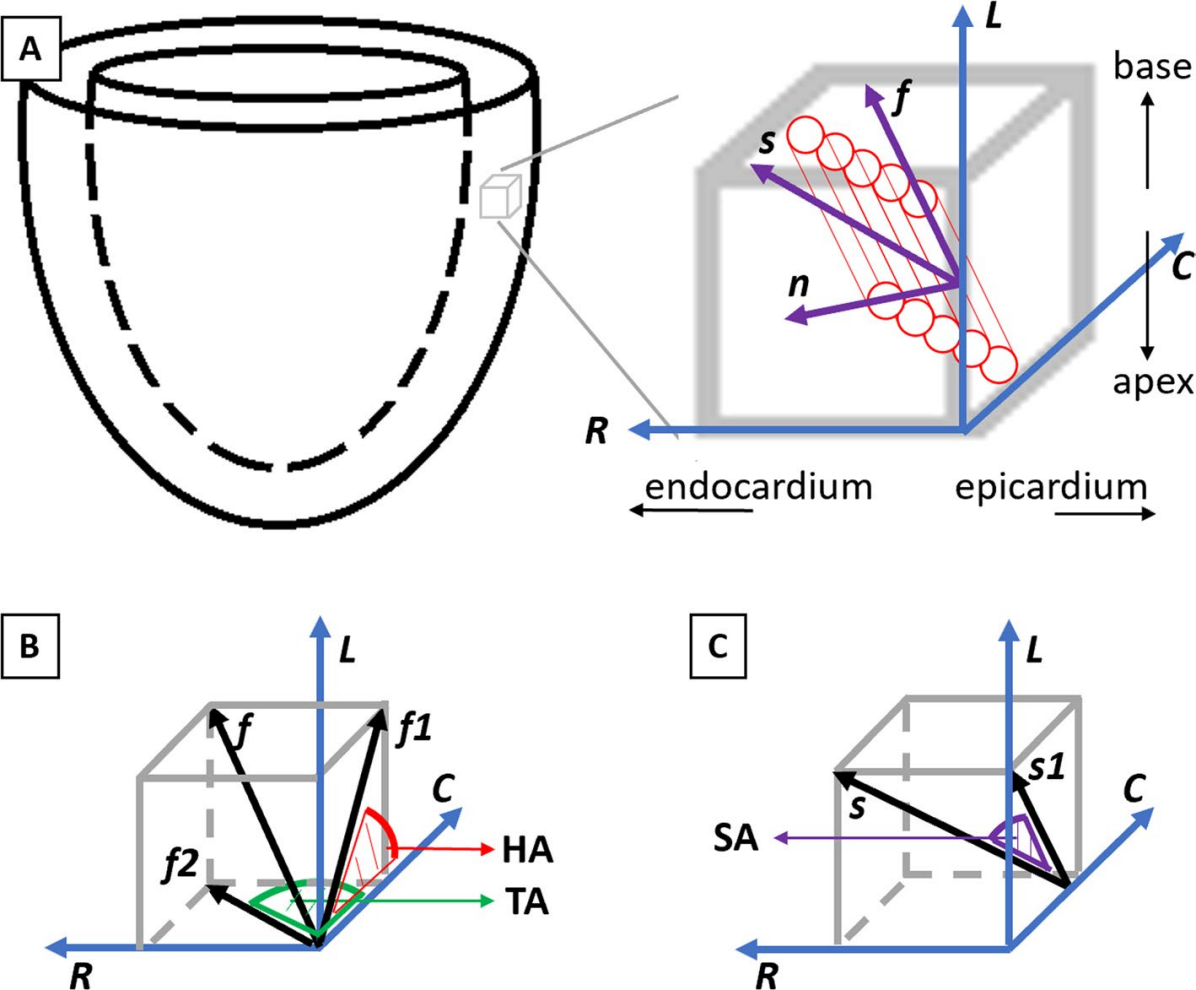
Definitions of parameters describing LV myocardial microstructure, helix angle (HA), transverse angle (TA) and sheetlet angle (SA). **a** Myocytes orientations (**f**) are depicted as cylinders, while sheetlets are depicted as groups of cylinders. The sheet direction (**s**) is perpendicular to **f** in the sheetlet plane, and the normal direction (**n**) is normal to the sheetlet. **L—**direction, **C—**circumferential direction, **R—**radial direction. **b** HA was defined as the angle between the projection of **f** on the local tangent **L–C** plane (**f**1) and C, while TA was defined as the angle between the projection of F on the **R–C** plane (**f**2) and C. **c** SA was defined as the angle between **s** and the projection of **s** on the **L–C** plane (**s1**)

Myocardial myocyte helix angle (HA) was defined as the angle between the projection of myocyte direction (***f***) onto the local longitudinal–circumferential plane and the circumferential axis (Fig. 3). Helix angle of the healthy geometry was set to vary linearly from + 60$^\circ$ and − 60 $^\circ$ from the endocardium to the epicardium (Streeter et al. 1969). We assumed that the healthy geometry could be transformed into diseased geometries via a homogeneous deformation, increased wall thickness for HCM and LV dilation for DCM. We further assumed that myocyte orientations would undergo the same transformation, to be realigned according to the deformation. Thus, a dilation in LV diameter would stretch the myocardium circumferentially and reduce the helix angle magnitudes via a cosine rule:

3 $$\begin{array}{*{20}c} {\frac{{\cos \left( {\theta_{{{\text{healthy}}}} } \right)}}{{\cos \left( {\theta_{{{\text{diseased}}}} } \right)}} = \frac{{L_{{\text{longi,healthy}}} /L_{{\text{circ, healthy}}} }}{{L_{{\text{longi, diseased}}} /L_{{\text{circ,diseased}}} }} = \frac{{D_{{{\text{diseased}}}} }}{{D_{{{\text{healthy}}}} }}} \\ \end{array}$$

where $\theta$ was the helix orientation, L was the longitudinal or circumferential length in the healthy or diseased LV model as indicated by the subscripts, and D was the diameter of the healthy or diseased LV as indicated by the subscript. Based on this, myocyte helix orientation of HCM was calculated to be + 60$^\circ$ to −51.4 $^\circ$ from endocardium to epicardium, and for DCM was + 49.7$^\circ$ and − 51.1$^\circ$. The helix angles in HCM and DCM were close to those in healthy LV after remodeling, which was consistent with the helix angle measurements in (Nielles-Vallespin et al. 2017).

Myocardial sheetlet angle (SA) was the angle between the sheetlet (***s***) direction and its projection on the local longitudinal–circumferential plane (Fig. 3) at the load-free state. We investigated three SA in the healthy LV, 0$^\circ$, 18$^\circ$ and 48$^\circ$, to gauge its effects on the LV functions. These were approximate from (Nielles-Vallespin et al. 2017), where 18$^\circ$ was the average diastolic SA for healthy and DCM LVs, while 48$^\circ$ was that for HCM LV.

Myofiber transverse angle (TA) was the angle between the projection of myocyte (F) direction on the local radial—circumferential plane and the circumferential axis (Fig. 3). Vendelin et al. found that TA near to 10$^\circ$ provided the best cardiac function efficiency (Vendelin et al. 2002). Here, we tested four transverse angles, 0$^\circ$, 10$^\circ$, 20$^\circ$ and 30$^\circ$. The spatial variation in transverse angles, $\delta$, was modeled as follows, as proposed by Vendelin et al.,

4 $$\begin{array}{*{20}c} {\delta = {\text{transverse }}\,{\text{angle}}*\omega \left( {1 - \left( {1 - 2\xi } \right)^{2} } \right)} \\ \end{array}$$

where $\xi$ was the normalized distance linearly ranging from −1 in the endocardium surface to 1 in the epicardial surface, $\omega$ was a linearly varied coefficient ranging from 0.5 at the base to −1 at the apex.

## Results

### Pressure–volume loop from FEM

**Fig. 4:**
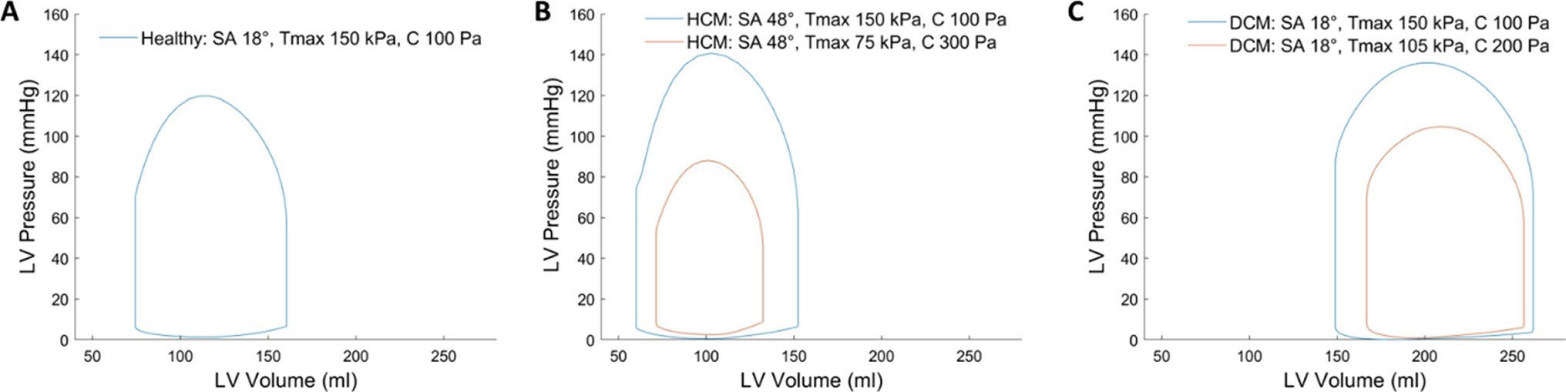
**a** Pressure–volume (PV) loop of healthy LV geometry when the diastolic sheetlet angle (SA) was 18 $^\circ$, myocardial contractility ${T}_{\mathrm{max}}$ was 150 kPa and global myocardial stiffness C was 100 Pa, **b** PV loop of HCM LV geometry when the diastolic SA was 48 $^\circ$, and ${T}_{\mathrm{max}}$ and C were the same as in the healthy geometry, or when ${T}_{\mathrm{max}}$ was reduced and C was increased, **c** PV loop of DCM LV geometry when the diastolic SA was 18 $^\circ$, and ${T}_{\mathrm{max}}$ and C were the same as in the healthy geometry, or when ${T}_{\mathrm{max}}$ was reduced and C was increased. All the transverse angle and shear stiffness ${B}_{{\varvec{n}}{\varvec{s}}}$ were 10 $^\circ$ and 15.0, other parameters are fixed as Tables 4 and Table 5

The pressure–volume (PV) loops of the FEM results are shown in Fig. 4. In the normal healthy LV with ${B}_{{\varvec{n}}{\varvec{s}}}=15.0$, peak TA = 10$^\circ$, diastolic SA = 18$^\circ$, global stiffness $C=100 \mathrm{Pa}$, and active tension ${T}_{\mathrm{max}}=150 \mathrm{kPa}$. Its ejection fraction (EF) was 53.8%, its peak systolic pressure was 119.7 mmHg, and its end-diastole to end-systole global longitudinal and circumferential strains were − 0.140 and − 0.203 (spatially averaged, with end-diastole as the zero-strain reference). These outcomes were close to a typical healthy heart (Kleijn et al. 2015; Muraru et al. 2014). For hypertrophied LVs with associated adjustments of diastolic sheetlet and helix angle, which represents HCM, EF was 60.8% when contractility and passive stiffness were maintained at that of the healthy LV (${T}_{\mathrm{max}}$ = 150 kPa and *C* = 100 Pa), and EF was reduced to 46.1% when contractility was reduced and passive stiffness was increased to more realistically reflect disease physiology (${T}_{\mathrm{max}}$ = 75 kPa, *C* = 300 Pa). For the dilated LV with associated sheetlet and helix angles adjustments, which represents DCM, EF was 43.2% when contractility and passive stiffness were maintained at that of the healthy LV (${T}_{\mathrm{max}}$ = 150 kPa and *C* = 100 Pa), but was reduced to 34.9% when contractility was reduced and stiffness increased (${T}_{\mathrm{max}}$ = 105 kPa, *C* = 200 Pa). These results demonstrate that FEM can be flexibly applied to emulate disease features.

### Effects of diastolic myocardial sheetlet angle and transverse angle on sheetlet sliding function

**Fig. 5:**
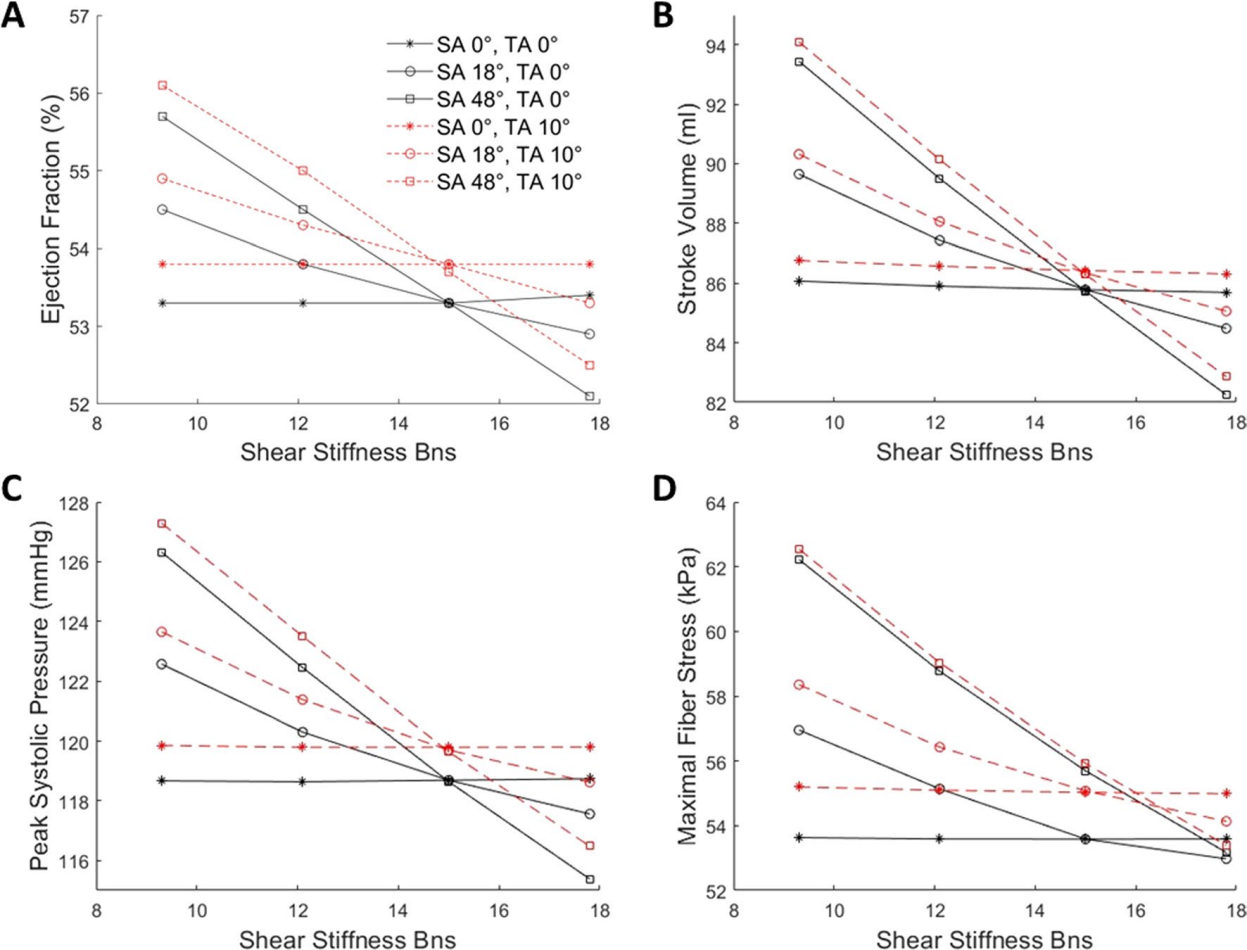
Effects of sheetlet sliding on LV functions and biomechanics in the healthy LV geometry at various sheetlet and transverse angles. Results on **a** ejection fraction, **b** stroke volume, **c** peak systolic pressure and **d** maximal fiber stress during systole were presented. ${T}_{\mathrm{max}}$ was 150 kPa and C was 100 Pa for all cases

We tested the effects of sheetlet sliding when diastolic sheetlet angle was 0$^\circ$ or 18$^\circ$ in the healthy geometry. Results are shown in Fig. 5 (and Table 1). Here, the horizontal axis was the ${B}_{{\varvec{n}}{\varvec{s}}}$ stiffness parameter where low ${B}_{{\varvec{n}}{\varvec{s}}}$ signifies greater extent of sheetlet sliding, while the vertical axis was a cardiac function or biomechanics function indicator. Where cardiac function varied substantially with ${B}_{{\varvec{n}}{\varvec{s}}}$ and the gradient of the plot was significant, this indicates that sheetlet sliding had a significant effect on function, and vice versa.

**Table 1:** Gradients of the relationships between shear stiffness ${B}_{{\varvec{n}}{\varvec{s}}}$ and various LV functions in the healthy LV geometry at various sheetlet and transverse angles

SA ($^\circ$)	TA ($^\circ$)	Gradients with regards to shear stiffness ${B}_{{\varvec{n}}{\varvec{s}}}$			
		EF (%)	SV (ml)	Peak systolic pressure (mmHg)	Maximal fiber stress (kPa)
0	0	0.002	− 0.044	0.008	− 0.004
18	0	− 0.180	− 0.610	− 0.590	− 0.468
48	0	− 0.418	− 1.316	− 1.286	− 1.064
0	10	− 0.006	− 0.054	− 0.008	− 0.026
18	10	− 0.160	− 0.532	− 0.492	− 0.414
48	10	− 0.422	− 1.322	− 1.270	− 1.076

Results showed that when diastolic sheetlet angle was 18$^\circ$, the physiologic configuration, sheetlet sliding had a modest impact on cardiac function and tissue stress in the myocyte direction, where EF, stroke volume, systolic pressure and myocardium stress in the myocyte direction were higher with more sliding. However, when diastolic sheetlet angle was 0$^\circ$ (sheetlets aligned with LV wall), these effects of sheetlet sliding disappeared, and sliding no longer had an influence on cardiac function or tissue stress. Conversely, when sheetlet angle was 48°, the configuration found for HCM LVs, the effects of sheetlet sliding were substantially amplified. Results thus indicated that sheetlet sliding can enable better cardiac function but produced higher myocardial stresses, and for this to occur, sheetlet angles must not be close to zero, i.e., the sheetlets must not be well-aligned with the myocardial wall plane. When the shear strain associated with sheetlet sliding, ${{\varvec{E}}}_{{\varvec{n}}{\varvec{s}}}$, was extracted from a mid-wall, mid-ventricular location of the healthy LV, the maximal ${{\varvec{E}}}_{{\varvec{n}}{\varvec{s}}}$ was observed to increase with sheetlet angle (${{\varvec{E}}}_{{\varvec{n}}{\varvec{s}}}$ = 0.01, 0.23 and 0.31 for sheetlet angle = 0°, 18° and 48°, respectively, at ${B}_{{\varvec{n}}{\varvec{s}}}$ = 9.3). This shows that greater extent of sheetlet sliding indeed occurred to give the functional advantages, confirming that sheetlet sliding was the mechanism for these advantages.

In Fig. 5, we also tested the effects of transverse angle. While a greater transverse angle was found to enhance cardiac function and elevate myocardial stresses, it had only very minor influence on the effects of sheetlet sliding, as the plots retained very similar gradient at both transverse angles (also shown in Table 1). We further conducted tests of various transverse angles, as shown in Appendix Fig. 10. Results showed that the optimal transverse angle was around 10$^\circ$ for the healthy and DCM geometries, which corroborated earlier findings (Vendelin et al. 2002), but the effect of transverse angle was generally weak, where cardiac function changed only slightly with transverse angle. With a hypertrophic LV wall, the effects of transverse angle were even weaker.

### Effects of helix angles on LV Functions and sheetlet sliding

**Fig. 6:**
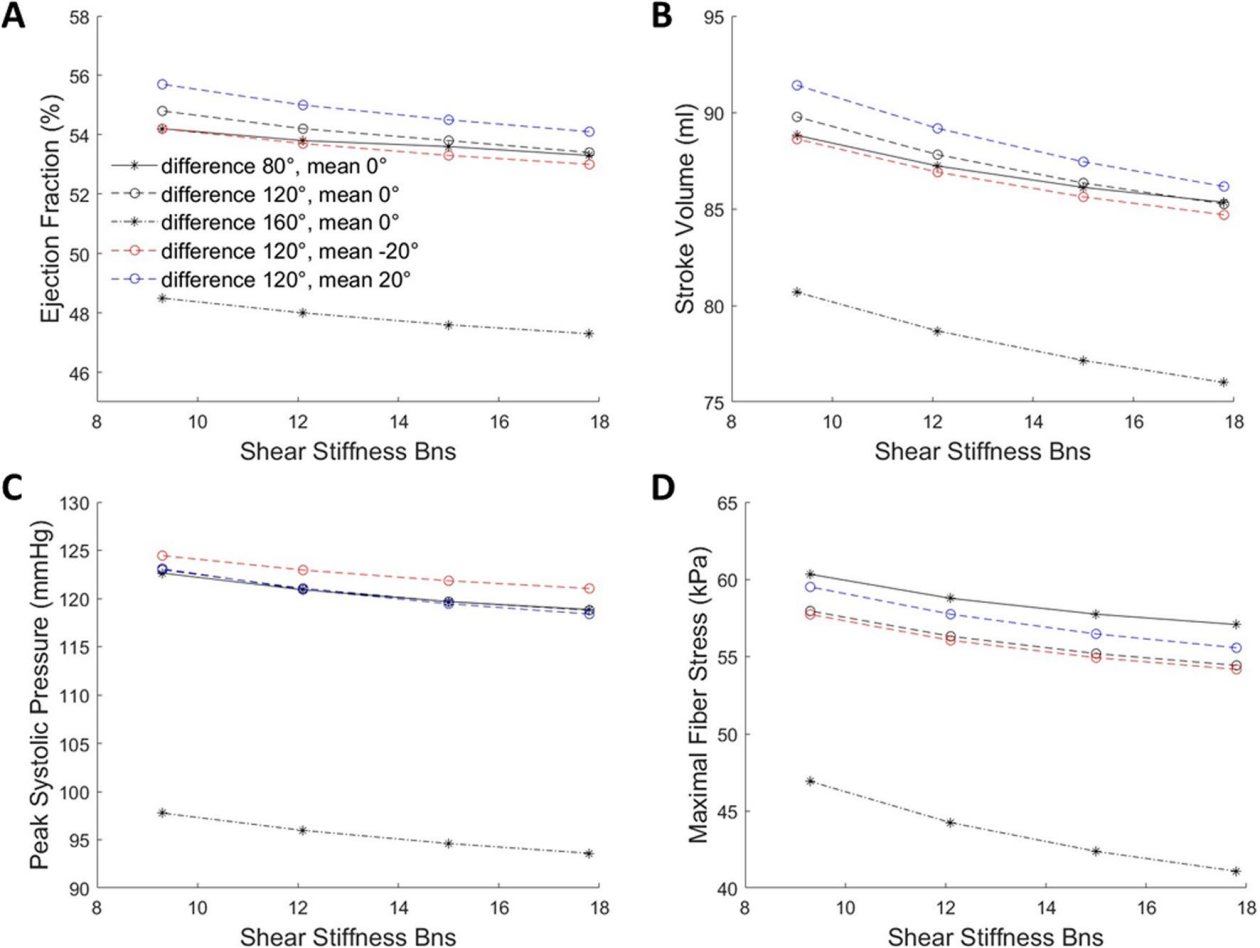
Effects of sheetlet sliding on LV function and biomechanics in the healthy LV geometry at various helix angle configurations (transmural angle differences and transmurally mean angle). Results on **a** ejection fraction, **b** stroke volume, **c** peak systolic pressure and **d** maximal fiber stress during systole were presented. ${T}_{\mathrm{max}}$ was 150 kPa, and C was 100 Pa for all cases

We further investigated how different helix angle configurations will impact the sheetlet sliding function, using the healthy and HCM LVs (SA 48°, EDP 7 mmHg). Using the model of linear endo-to-epi helix angle variation, we investigated various cases of transmural helix angle difference (epi-to-endo difference in helix angle) and transmurally averaged helix angle. Results are shown in Fig. 6 for healthy LV, and the gradients parameters versus Bns are quantified in Table 2 for healthy and HCM LVs. These results showed that only helix angle configuration has a minor but non-negligible influence on sheetlet sliding effects (represented by the gradient of various functional parameters with ${B}_{{\varvec{n}}{\varvec{s}}}$). An overall optimal point was observed close to the epi-to-endo helix angle configuration of − 40° to + 80°, while departure from this configuration decreases such gradients.

**Table 2:** Gradients of the relationships between shear stiffness ${B}_{{\varvec{n}}{\varvec{s}}}$ and various LV functions in the healthy and HCM LV geometries at various helix angle configuration

Transmural angle difference ($^\circ$)	Transmurally averaged angle ($^\circ$)	Gradient with respect to shear stiffness ${B}_{{\varvec{n}}{\varvec{s}}}$			
		EF (%)	SV (ml)	Peak systolic pressure (mmHg)	Maximal fiber stress (kPa)
*Healthy LV*					
80	0	− 0.104	− 0.41	− 0.446	− 0.384
120	− 20	− 0.144	− 0.462	− 0.400	− 0.420
120	− 10	− 0.150	− 0.492	− 0.422	− 0.406
120	0	− 0.160	− 0.532	− 0.492	− 0.414
120	10	− 0.172	− 0.572	− 0.526	− 0.438
120	20	− 0.188	− 0.618	− 0.554	− 0.464
160	0	− 0.140	− 0.550	− 0.490	− 0.686

Transmural angle ($^\circ$)	Average angle ($^\circ$)	Gradients with respect to shear stiffness ${B}_{{\varvec{n}}{\varvec{s}}}$			
		EF (%)	SV (ml)	Peak systolic pressure (mmHg)	Maximal fiber stress (kPa)
*HCM LV*					
80	0	− 0.344	− 1.128	− 1.346	− 0.476
120	− 20	− 0.502	− 1.298	− 1.374	− 0.464
120	0	− 0.592	− 1.550	− 1.564	− 0.530
120	20	− 0.702	− 1.806	− 1.770	− 0.596
160	0	− 0.662	− 1.776	− 1.650	− 0.576

### Effects of cardiomyopathy remodeling on LV functions and sheetlet sliding

**Fig. 7:**
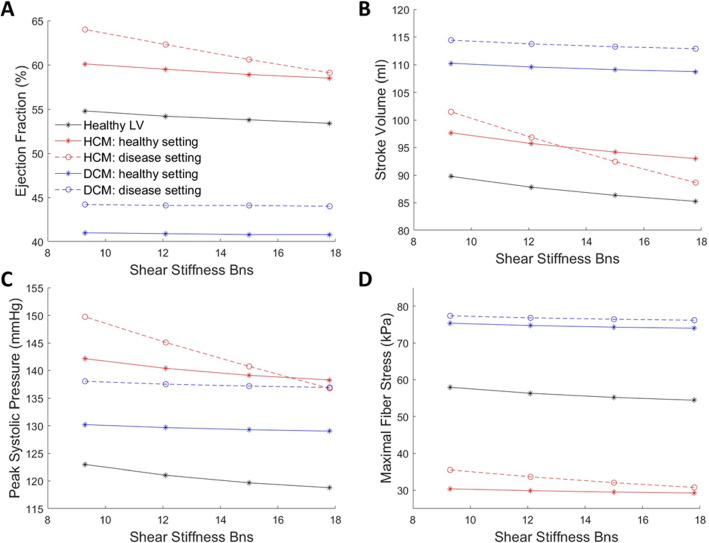
Effects of sheetlet sliding on LV function and biomechanics for healthy, wall-hypertrophied (HCM) and chamber-dilated (DCM) LV geometries. Results on **a** ejection fraction, **b** stroke volume, **c** peak systolic pressure and **d** maximal fiber stress during systole were plotted. In “HCM: healthy setting”, and “DCM: healthy setting” cases, FE simulation settings were the same as the “Healthy LV” case except the LV geometry. In “HCM: disease setting” case, the helix angles were 60$^\circ$ to − 51.4$^\circ$, the diastolic sheetlet angle was 48$^\circ$ and EDP was increased by 40% to 7 mmHg. In “DCM: disease setting” case, the helix angles were 49.7$^\circ$ to − 51.1$^\circ$, the sheetlet angle was 18$^\circ$ and EDP was increased by 40% to 7 mmHg. Transverse angle was 10$^\circ$ for all cases

We performed sensitivity analysis to understand how LV geometry and physiological factors will affect the above sheetlet sliding effects, focusing on factors relevant to cardiomyopathy remodeling. Results are shown in Fig. 7 and Table 3.

**Table 3:** Gradients of the relationships between shear stiffness ${B}_{{\varvec{n}}{\varvec{s}}}$ and various LV functions in HCM and DCM at different settings

LV geometry	FE simulation settings	Gradient			
		EF (%)	SV (ml)	Peak systolic pressure (mmHg)	Maximal fiber stress (kPa)
Normal	Healthy settings	− 0.160	− 0.532	− 0.492	− 0.414
HCM	Healthy settings	− 0.194	− 0.552	− 0.454	− 0.134
HCM	Healthy settings, but EDP increased from 5 to 7 mmHg	− 0.208	− 0.576	− 0.488	− 0.142
HCM	Healthy settings, but epi:endo helix angle configuration changed from − 60°:60° to − 51.4°:60^o^	− 0.190	− 0.546	− 0.478	− 0.130
HCM	Healthy settings, but diastolic sheetlet angle changed from 18° to 48^o^	− 0.562	− 1.494	− 1.488	− 0.524
HCM	Disease settings	− 0.572	− 1.508	− 1.526	− 0.556
DCM	Healthy settings	− 0.016	− 0.178	− 0.140	− 0.160
DCM	Disease settings	− 0.018	− 0.180	− 0.130	− 0.140

Firstly, where only the LV geometry was changed, while all other physiological FE settings (helix angle configuration, sheetlet angle, EDP, stiffness and contractility) were retained at healthy LV settings, the influence of sheetlet sliding on cardiac function was slightly improved in the HCM LV, where gradients of functional parameters versus ${B}_{{\varvec{n}}{\varvec{s}}}$ did not vary much from that in the healthy LV simulation, except for gradient of myocardial stresses versus ${B}_{{\varvec{n}}{\varvec{s}}}$, which was significantly decreased likely because to the HCM LV having thicker walls and thus, reduced stresses. In the DCM LV, however, gradients of functional parameters were drastically decreased (by 61–90%). These suggest that the DCM geometry inhibited effects of sheetlet sliding, but the HCM geometry did not.

Next, we analyzed HCM and DCM simulations where both LV geometry and physiological FE settings were tuned to disease conditions. We observed that DCM simulations with diseased FE settings did not have significant difference from DCM simulations with healthy FE settings. This suggested that in DCM, LV geometry was the dominant factor determining the reduced effects of sheetlet sliding, rather than physiological factors.

On the other hand, for HCM simulations with diseased FE settings, the influence of sheetlet sliding on cardiac function was significantly increased. For example, the stroke volume versus ${B}_{{\varvec{n}}{\varvec{s}}}$ gradient for the HCM with disease settings case (gradient = − 1.508) increased by 174% from the HCM simulations with healthy FE settings (gradient = − 0.552) and increased 183% from the healthy LV simulations (gradient = − 0.532). From the above analysis, it was obvious that the HCM geometry alone cannot account for this difference. As such, we performed simulations with the HCM geometry under the healthy LV FE settings, but with a single physiological parameter varied to disease conditions, to understand which parameter was responsible for the drastic change to sheetlet sliding effects. We investigated EDP, helix angle, and sheetlet angle for this. Results in Table 3 showed that it was the change of sheetlet angle from 18° to 48° that was mainly responsible for the drastic change to sheetlet sliding effects, while changes to helix angle and EDP only caused minor changes.

We further noted that the changing sheetlet angle from 18° to 48° increased gradients (of functional parameters versus ${B}_{{\varvec{n}}{\varvec{s}}}$) by 148%-164% in the healthy LV geometry, but it increased gradients by 171–291% in the HCM LV geometry. This suggested an intricate interplay between two factors: sheetlet angle and HCM geometry, which jointly enabling greater effects of sheetlet angle in enhancing cardiac function.

In Appendix Figs. 11 and 12 and Appendix Table 7, we further tested the effects of altering myocardial contractility ${T}_{\mathrm{max}}$ and the global myocardial stiffness coefficient *C* on the hypertrophied and dilated LV geometries. Results show that increasing contractility or stiffness coefficient generally caused a minor increase in the effects of sheetlet sliding. Thus, reduced contractility and altered overall myocardial stiffness during cardiomyopathy would not significantly affect sheetlet sliding mechanisms.

### Effects of sheetlet sliding on myocardial stresses and strains

**Fig. 8:**
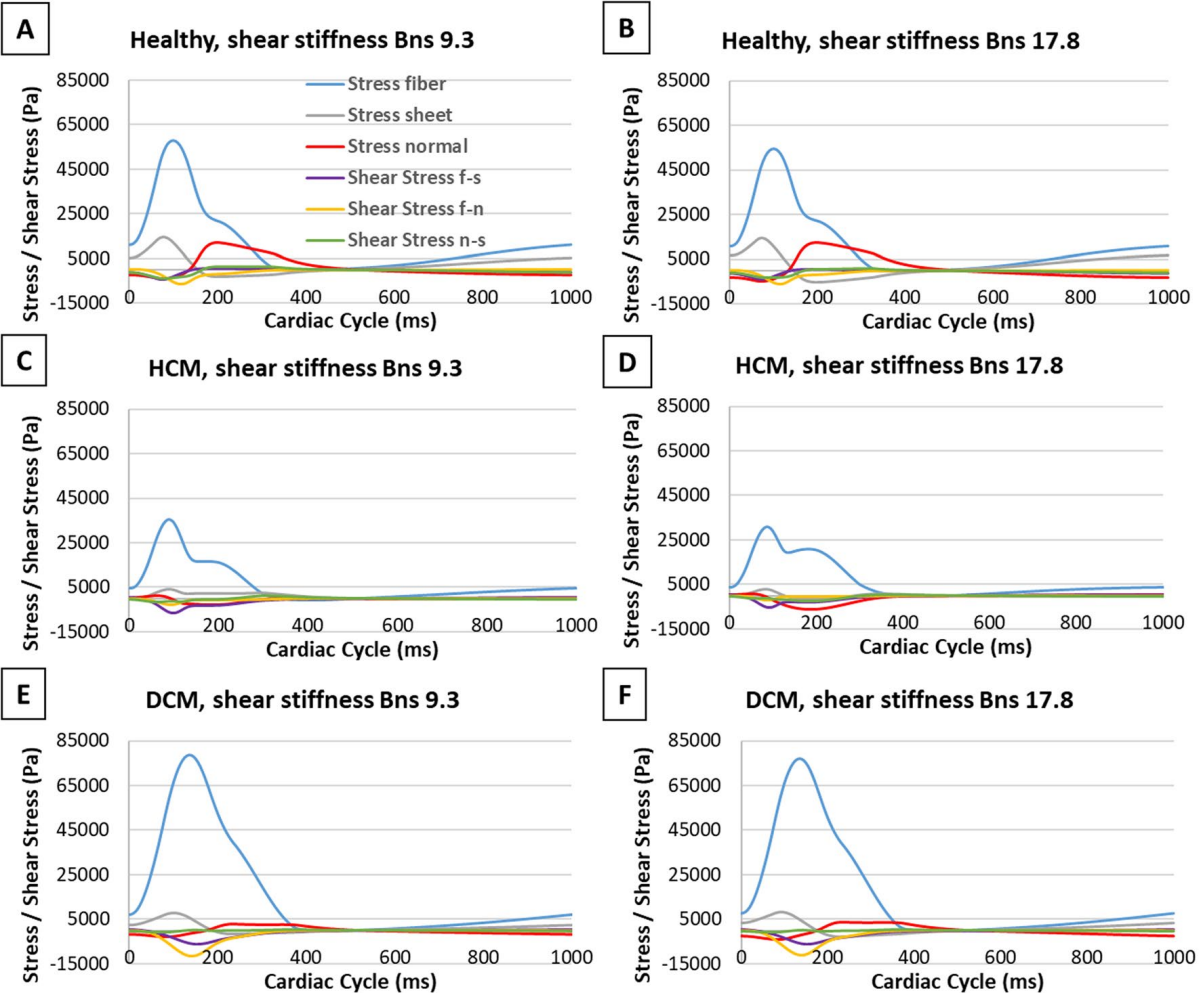
Various myocardial stress components of different LV geometries during the cardiac cycle. Results are shown for **a**, **b** healthy LV geometry, **c**,** d** HCM LV geometry with disease settings, and **e**, **f** DCM LV geometry with disease settings, for **a**, **c**, **e** ${B}_{{\varvec{n}}{\varvec{s}}}=9.3$ and **b**, **d**, **f** ${B}_{{\varvec{n}}{\varvec{s}}}=17.8$. Diastolic sheetlet angle was 18$^\circ$ for healthy and DCM LV geometries, and 48$^\circ$ for HCM LV geometry

Figure 8 shows the waveforms of various stress components over the cardiac cycle. The peak values of each stress component are quantified in appendix Fig. 14. Here, we found that shear stiffness ${B}_{{\varvec{n}}{\varvec{s}}}$ affected the magnitude of the peak stress in the “fiber” or myocyte direction the most. Peak fiber direction stress increased by 3.5 kPa and 4.7 kPa and 1.6 kPa in healthy, HCM and DCM LV. This meant that when sheetlet sliding increased, more stresses were concentrated in the fiber direction. Changing ${B}_{{\varvec{n}}{\varvec{s}}}$ only caused small magnitude changes to stresses in other directions, suggesting that the occurrence of sheetlet sliding did not alter these other stresses much. In some directions, sheetlet sliding actually reduced stresses, for example in the sheet and normal directions in the DCM LV and in the normal direction in the healthy LV.

**Fig. 9:**
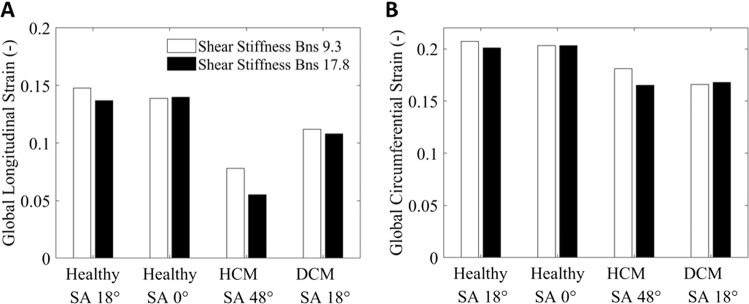
Magnitudes of **a** peak global longitudinal strain and **b** peak global circumferential strain for healthy, HCM and DCM LV geometries, at various sheetlet angle (SA) as indicated, and at ${B}_{{\varvec{n}}{\varvec{s}}}$ of 9.3 and 17.8. Transverse angles, myocardial contractility and global stiffness **c** were fixed at 10$^\circ$, 150 kPa and 100 Pa, respectively

Figure 9 presented the magnitudes of peak longitudinal strain and peak circumferential strain when the shear stiffness ${B}_{{\varvec{n}}{\varvec{s}}}$ were 9.3 and 17.8 for different geometries. Here, lower ${B}_{{\varvec{n}}{\varvec{s}}}$ led to elevated peak strains, suggesting that the sheetlet sliding enhanced the deformability of the myocardium, and this affected longitudinal strains more than circumferential strains. Similar to other cardiac function parameters, when sheetlet angle was 0°, allowing sheetlet sliding had very little effects on peak strains, but such effects were amplified with greater sheetlet angles. Comparing normal LV to diseased geometries, sheetlet sliding similarly had greater effects on strains in the HCM geometry and smaller effects in the DCM geometry.

## Discussions

Myocardial sheetlet sliding is thought to be an important dynamic mechanism that enables large ventricular tissue radial strains and wall thickening during systole (Costa et al. 1999; Nielles-Vallespin et al. 2017). The micro-structural reorganization of the sheetlets during contraction is thought to be the bridging mechanism to reach high strains. However, the effects of the sheetlet sliding to cardiac function are not understood. Here, we tested the hypothesis that sheetlet sliding also results in better cardiac output, by modeling sheetlet sliding as a reduced shear stiffness in the ***n-s*** and ***s–n*** directions, and through our finite element modeling, quantified the advantages that sheetlet sliding could offer to cardiac function, for normal and cardiomyopathy hearts. Since our modeling was limited to an assumption of reduced shear stiffness and has no consideration of the myocardium tissue micro-architecture and micro-dynamics, our investigation did not touch on how sheetlet sliding could enable large strains, but merely addressed the question of how much cardiac function advantages could be provided by sheetlet sliding by virtue of reduced shear stiffness.

Our first observation was that a nonzero diastolic sheetlet angle was necessary for sheetlet sliding to have an effect, and the larger the sheetlet angle (until angle of 45°), the more advantages sheetlet sliding can provide. This was likely because the usual myocardial deformations could not engage the sliding deformation if sheetlets that were perfectly aligned to the myocardial longitudinal–circumferential plane, leading to no advantage to function. With nonzero diastolic sheetlet angle, some components of the sheetlet sliding shear deformation would contribute to longitudinal and circumferential strains (as can be shown with coordinate axes transformation), reducing the resistance of the myocardium to its cyclic deformations, and enhancing function. Theoretically, at the sheetlet angle of 45°, the maximum amount of sheetlet sliding shear deformation would be aligned with longitudinal/circumferential strains, and the effects of sheetlet sliding would be at its maximum.

Our second observation was that sheetlet sliding has a stronger effect on cardiac function in the hypertrophied LV, a modest effect in the normal LV, and a weaker effect in the dilated LV. In the DCM LV, our results showed that it was the dilated geometry that inhibited sheetlet sliding effects. We speculate that this was due to the lower relative wall thickness (ratio of wall thickness to chamber diameter) in dilated hearts, effectively representing an LV with thinner walls, leading to reduced influence of wall stiffness on cardiac function. The mechanism by which sheetlet sliding enabled better cardiac function was that it reduced shear stiffness in specific directions, thus reducing resistance to deformation. In DCM, where wall stiffness was not as important, it logically followed that sheetlet sliding effects are weaker.

In the HCM LV, the hypertrophied LV had additional myocardial mass that led to a larger overall stiffness, which required increased energy burden to deform, thus causing increased resistance to deformation. Sheetlet sliding helped to reduce this deformational resistance and would thus have a stronger effect in HCM hearts. However, the most important factor that allowed strong sheetlet sliding mechanisms in HCM hearts was that the sheetlet angle was close to 45° (Ferreira et al. 2014; Nielles-Vallespin et al. 2017). As discussed above, this sheetlet angle configuration maximizes the effects of sheetlet sliding due to an effective transfer of sheetlet sliding shear strain to longitudinal/circumferential strains. Thus, our study results suggested that both sheetlet angle orientation and geometry remodeling during cardiomyopathy were important factors altering sheetlet sliding mechanisms and cardiac function.

Our studies also found several other factors have minor impact on the sheetlet sliding’s influence on cardiac function, including helix angle configuration, transverse angle magnitude, contractility and tissue stiffness. However, to accurately capture sheetlet sliding effects, it seems important that these factors are included in FE models to fully capture sheetlet sliding physics.

Further, we observed that the advantages of sheetlet sliding to cardiac function were balanced by an increase in myocardial stresses. When function was increased due to sliding, there was a redistribution of stresses, and a greater magnitude of stress in the myocyte direction. This could be related to the coupling between fiber and cross-fiber stretches, which also observed by (Nordsletten et al. 2021). Excessive stress is likely to be detrimental to cardiac health. For example, excessive myocardial stress during hypertensive heart failure and myocardial infarction leads to harmful cardiac remodeling and cardiac failure (deSimone et al. 1992; Paulus Walter and Dal Canto 2018). Therefore, we speculate that on top of being an adaptation to enhance cardiac function, sheetlet sliding was also a compromise and balance between enhanced function and elevated myocardial stresses.

One interesting result of our simulations was that in our simulation of the normal heart, the sheetlet angle changed by only 8° between systole and diastole (sheetlet angle mobility of 8°), whereas Nielles-Vallespin et al. had measured sheetlet angle mobility to be 47° via DT-MRI (Nielles-Vallespin et al. 2017). In our simulations, the myocardial sheetlets were assumed to be fixed to the macroscopic tissues, such that the sheetlet angle mobility was caused solely by the macroscopic tissue deformations, and sheetlets were assumed to undergo no microscopic tissue reorganization that would cause further reorientation. This thus brings to light that tissue architectural reorganization must have occurred during myocardial contraction to enable this large measured sheetlet angle mobility. We double checked this idea in appendix section A6, where we performed idealized calculation of diastole-to-systole sheetlet angle changes in a small myocardial cube with the no-tissue-reorganization assumption above, and found that our FE sheetlet angle mobility was close to the idealized calculations. In the same section, we further calculated that, in order to achieve a sheetlet angle mobility of 45°, a radial stretch of 333% was needed, which was much larger than the 64% peak radial strain reported by (Nielles-Vallespin et al. 2017). These thus corroborated with the notion that some significant tissue reorganization is likely to be occurring during the usual diastole-to-systole contractile deformation that enhances sheetlet angle mobility, above those caused by continuum deformation. It is currently unclear what type of tissue reorganization may be occurring, and further studies seem much warranted. However, we noted that the myocardial microstructure is complex and likely capable of such reorganization; for example, Kung et al. found that there are diverse populations of sheetlets in myocardium (Kung et al. 2011) and MRI sheetlet angles measurements can only provide the average sheetlet angle over a voxel space.

Overall, although this study revealed the effects of sheetlet sliding on cardiac function and the dependencies of sheetlet sliding on physiological parameters, many further questions arose from it, the answer to which required future work. We suggest that it would be very useful for such future work to image the microstructural changes of myocardium during the contraction or relaxation process to understand microstructural characteristics of sheetlet sliding, and to compare such microstructural characteristics between healthy and cardiomyopathic myocardium. Such characterization will also be useful for formulating more accurate constitutive models of sheetlet sliding.

## Limitations

There are several limitations to our work. Firstly, we conducted the study based on only one healthy volunteer dataset, and the helix angle and sheetlet angle were not case-specific. Our assumption for diseased LV helix angle configurations was also not validated. Future work is thus needed to evaluate more realistic helix angle configurations.

Secondly, HCM and DCM LV geometries were not obtained directly from patients, but were morphed and derived from the healthy LV. However, our approach had the further advantage that it allowed us to fix the overall shape of the heart and isolate wall thickness and chamber dilation as variables when testing the effects of LV shape changes on sheetlet sliding.

Thirdly, the lumped-parameter circulatory model was fixed at that of the healthy individual and was not modified to reflect changes in disease. The magnitude of sheetlet sliding effects on function could change when patient-specific lumped-parameter models were used, but we did not expect the overall conclusions to be altered.

Fourthly, the shear stiffnesses we adopted as being relevant to sheetlet sliding in our simulations were based on the Fung-type model matched to single data points from Sommer et al. (2015) rather than completely matching the stress–strain curves. Our results are thus limited specifically to this material stiffness settings and have not been explored with alternative constitutive material laws.

Fifthly, our method to obtain the load-free geometry is an approximate one, designed to achieve a specific end-diastolic pressure and volume, rather than a more comprehensive match of the end-diastolic LV anatomy (e.g., thickness, diameter and length). With our current approach, when the load-free geometry was loaded to the end-diastolic state, errors were approximately 3.2% for long axis length, 7.3% for wall thickness, and 0.7% for diameter. However, since our inflated geometry was still reasonable physiological, study conclusion should not be affected.

Finally, the definition of the sheetlet angle was identical to its definition in the previous study (Nielles-Vallespin et al. 2017) only when the transverse angle was zero. However, the transverse angle was small in the myocardium (Lombaert et al. 2012) and its effects on the LV functions were quite limited from the study. Thus, we do not expect this approximation to have a substantial effect on our results.

## Conclusions

We performed a myocardial sheetlet sliding study using finite element simulation and investigated the influences of sheetlet angle, shear stiffness and hypertrophied and dilated geometry remodeling. We found that a nonzero diastolic sheetlet angle was essential for sheetlet sliding to have an effect on cardiac function, and that sheetlet sliding improved LV functions in terms of stroke volume, ejection fraction, and systolic pressure generation. We further found that this effect of sheetlet sliding was modest in the healthy LV, amplified in the wall-hypertrophied LV, but reduced in the dilated LV. However, when sheetlet sliding improved function, it also increased myocardial stress, elevating stress in the myocyte direction. We thus speculate that sheetlet sliding to be a tissue architectural adaptation to allow easier deformations of thick LV walls, so as to enhance function, but that it also represented a compromise and balance between function and tissue stress.

### Electronic supplementary material

Below is the link to the electronic supplementary material.

Supplementary file1 (ZIP 7202 kb)

## Figures and Tables

**Fig. 10:**
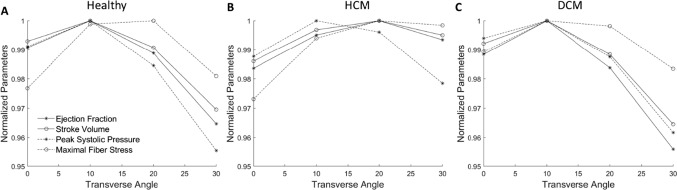
Effects of transverse angle on the LV function and biomechanics in the **a** healthy geometry, **b** HCM geometry and **c** DCM LV geometry. The sheetlet angles were 18$^\circ$, 48$^\circ$ and 18$^\circ$, for healthy, HCM and DCM LV geometries, respectively, and ${B}_{{\varvec{n}}{\varvec{s}}}$ was 15.0 for all cases. All the parameters were normalized to their maximal values

**Fig. 11:**
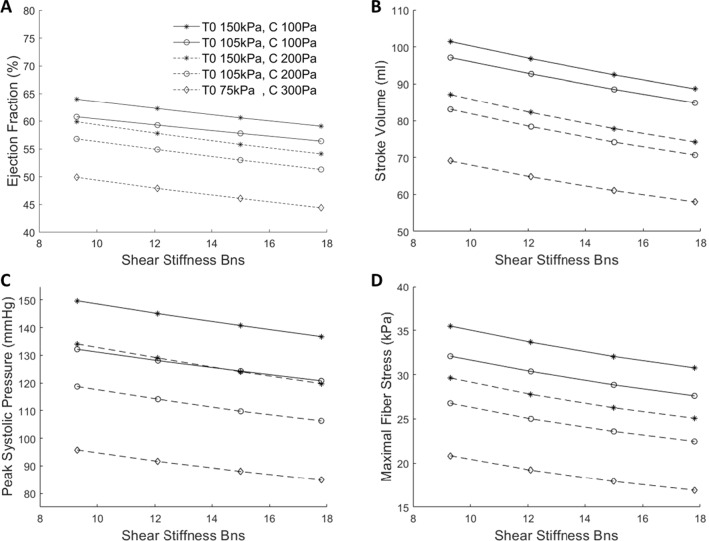
For the hypertrophic LV geometry, changing myocardial contractility and myocardial stiffness coefficient did not alter the influence of sheetlet sliding on LV cardiac function and biomechanical parameters, including **a** ejection fraction, **b** stroke volume, **c** peak systolic pressure and **d** maximal fiber stress

**Fig. 12:**
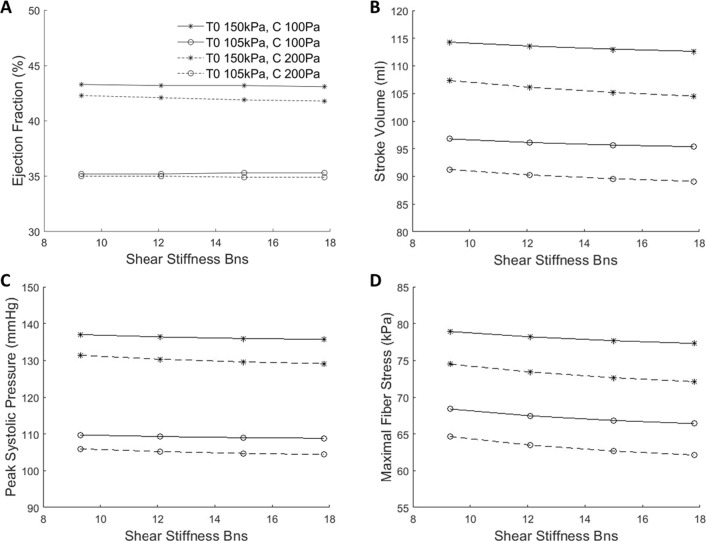
For the dilated LV geometry, changing myocardial contractility and myocardial stiffness coefficient did not alter the influence of sheetlet sliding on LV cardiac function and biomechanical parameters, including **a** ejection fraction, **b** stroke volume, **c** peak systolic pressure and **d** maximal fiber stress

**Fig. 13:**
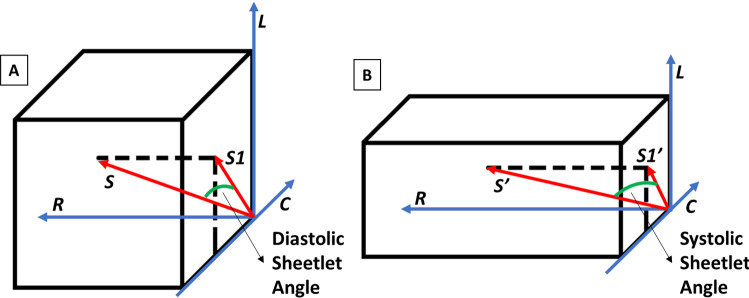
**a** Diastolic sheetlet angle between **S** and **S1**, and **b** systolic sheetlet angle between **S’** and **S1’** using a continuum material. **S** and **S’**: sheet direction; **S1** and **S1’**: projection of sheet direction on the local **L**–**C** wall tangent plane; **L**: longitudinal direction; **C**: circumferential direction; **R**: radial direction

**Fig. 14:**
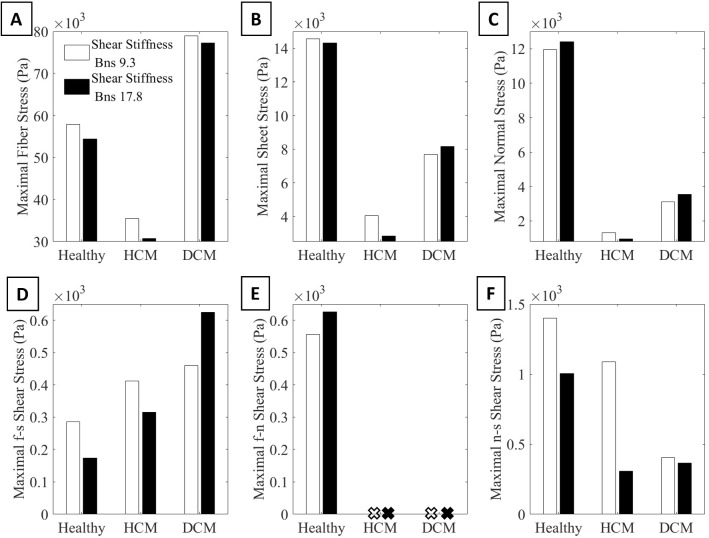
Bar plots of maximum tensile stress components with differing ${B}_{{\varvec{n}}{\varvec{s}}}$, corresponding to plots in Fig. 8. Crosses indicate values close to zero

## Appendix

### Section A1: Myocardial Guccione active tension model

The Guccione model (Guccione et al. 1993) was employed to describe myocardial active tension, which was based on a calcium activation model, where the active stress ${{\varvec{\sigma}}}_{{\varvec{a}}}$ was calculated as:

5 $$\begin{array}{*{20}c} {{\varvec{\sigma}}_{{\varvec{a}}} = T_{{{\text{max}}}} \frac{{{\text{Ca}}_{0}^{2} }}{{{\text{Ca}}_{0}^{2} + E{\text{Ca}}_{50}^{2} }}C_{t } {\varvec{f}} \otimes {\varvec{f}}_{0} } \\ \end{array}$$

where ${T}_{\mathrm{max}}$ was the maximum active tension, ${\mathrm{Ca}}_{0}$ was the peak calcium concentration, ${\varvec{f}}$ and ${{\varvec{f}}}_{0}$ were the local fiber directions in the current stage and the reference stage, ${E\mathrm{Ca}}_{50}$ was the calcium sensitivity which was dependent on sarcomere length and ${C}_{t}$ was the temporal variation coefficient. ${E\mathrm{Ca}}_{50}$ and ${C}_{t}$ were described as following equations:

6a $$\begin{array}{*{20}c} {E{\text{Ca}}_{50} = \frac{{({\text{Ca}}_{0} )_{\max } }}{{\sqrt {\exp \left( {B\left( {l - l_{0} } \right)} \right) - 1} }}} \\ \end{array}$$

6b $$\begin{array}{*{20}c} {C_{t} = \frac{1}{2}\left( {1 - \cos \omega } \right)} \\ \end{array}$$

6c $$\begin{array}{*{20}c} {\omega = \left\{ {\begin{array}{*{20}l} {\pi \frac{t}{{t_{0} }}} \hfill & {{\text{when}} \quad 0 \le t < t_{0} } \hfill \\ {\pi \frac{{t - t_{0} + t_{r} }}{{t_{r} }}} \hfill & {{\text{when }}\quad t_{0} \le t < t_{0} + t_{r} } \hfill \\ 0 \hfill & { {\text{when}} \quad t \ge t_{0} + t_{r} } \hfill \\ \end{array} } \right.} \\ \end{array}$$

where ${t}_{0}$ was the time to peak tension, which specifies how long it takes the myofibers to contract, and ${t}_{r}$ was the relaxation time. ${t}_{0}$ was specified according to measurements by (Mulieri et al. 1992), and depended on heart rate (HR), as follows:

7 $$\begin{array}{*{20}c} {t_{0} = - 0.96*HR + 243} \\ \end{array}$$

while $t_{r}$ was calculated as following equation:

8a $$\begin{array}{*{20}c} {t_{r} = ml + b} \\ \end{array}$$

8b $$\begin{array}{*{20}c} {l = \sqrt {{\varvec{f}}_{0} \cdot {\varvec{C}} \cdot {\varvec{f}}_{0} } l_{r} } \\ \end{array}$$

8c $$\begin{array}{*{20}c} {{\varvec{C}} = {\varvec{F}}^{T} \cdot {\varvec{F}}} \\ \end{array}$$

where *m* was the slope of the relaxation duration and *b* was the time-intercept of linear duration, and $l$ was the real time sarcomere length, and ${\varvec{C}}$ was the right Cauchy–Green deformation tensor calculated from the deformation tensor ${\varvec{F}}$ (Table

**Table 4:** Default fixed parameters for FE model (Shavik et al. 2018)

Parameters	Unit	Values
Fiber direction material stiffness,${B}_{{\varvec{f}}{\varvec{f}}}$	Unitless	29.8
Cross-fiber direction material stiffness, ${B}_{{\varvec{s}}{\varvec{s}}}$,${B}_{{\varvec{n}}{\varvec{n}}}$	Unitless	14.9
Shear mode material stiffness, ${B}_{{\varvec{f}}{\varvec{s}}}$,${B}_{{\varvec{f}}{\varvec{n}}}$	Unitless	19.2
Peak intracellular Ca concentration,$C{a}_{0}$	$\mathrm{\mu M}$	4.35
Maximum peak intracellular Ca concentration,${(C{a}_{0})}_{max}$	$\mathrm{\mu M}$	4.35
Parameter for isometric tension (sarcomere length relation), *B*	${\mathrm{\mu m}}^{-1}$	4.75
Sarcomere length at zero-active tension,${l}_{0}$	$\mathrm{\mu m}$	1.58
Relaxed sarcomere length,${l}_{r}$	$\mathrm{\mu m}$	1.85
Heart rate, HR	$\mathrm{beat}*{\mathrm{min}}^{-1}$	60
Time to LV peak tension,${t}_{0}$	$\mathrm{ms}$	185.5
Slope of linear relaxation duration (sarcomere length relation), *m*	$\mathrm{ms}*{\mathrm{\mu m}}^{-1}$	1049
Time-intercept of linear relaxation duration (sarcomere length relation), *b*	$\mathrm{ms}$	−1600

^4^)

### Section A2: Windkessel lumped-parameter model

The time-varying LA elastance model was described as:

9a $$\begin{array}{*{20}c} \begin{aligned} P_{{{\text{LA}}}} \left( t \right) & = e\left( t \right)*E_{{\text{es,LA}}} *\left( {V_{{{\text{LA}}}} \left( t \right) - V_{{\text{0, LA}}} } \right) \\ & \quad + \left( {1 - e\left( t \right)} \right)*A_{{{\text{LA}}}} *\left( {e^{{B_{{{\text{LA}}}} *\left( {V_{{{\text{LA}}}} \left( t \right) - V_{{\text{0,LA}}} } \right)}} - 1} \right) \\ \end{aligned} \\ \end{array}$$

9b $$\begin{array}{*{20}c} {e\left( t \right) = \left\{ {\begin{array}{*{20}c} {\frac{1}{2}\left( {\sin \left[ {\left( {\frac{\pi }{{t_{{\max ,{\text{LA}}}} }}} \right)t - \frac{\pi }{2}} \right] + 1} \right)} & {{\text{when }}\,\,\,0 < t \le \frac{3}{2}t_{{\max ,{\text{LA}}}} } \\ {\frac{1}{2}e^{{ - \frac{{t - \frac{3}{2}t_{{\max ,{\text{LA}}}} }}{{\tau_{{{\text{LA}}}} }}}} } & {{\text{when}}\,\, t > \frac{3}{2}t_{{\max ,{\text{LA}}}} } \\ \end{array} } \right.} \\ \end{array}$$

where ${E}_{\mathrm{es},\mathrm{LA}}$ was the LA end-systolic elastance, ${V}_{\mathrm{LA}}\left(t\right)$ and ${V}_{0,\mathrm{LA}}$ were the LV volume at any time and volume intercept of the end-systolic pressure–volume relationship (ESPVR), and ${A}_{\mathrm{LA}}$ and ${B}_{\mathrm{LA}}$ were parameters of the end-diastolic pressure–volume relationship (EDPVR), ${t}_{\mathrm{max},\mathrm{LA}}$ was the time point with the maximal LA elastance and ${\tau }_{\mathrm{LA}}$ was the LA relaxation time constant (Table

**Table 5:** Default fixed parameters for windkessel circulatory and LA elastance model, as obtained from (Shavik et al. 2018)

Parameters	Unit	Values
Aortic valve resistance, Rao	$$\mathrm{Pa}*\mathrm{ms}*{\mathrm{ml}}^{-1}$$	6500
Mitral valve resistance, Rmv	$$\mathrm{Pa}*\mathrm{ms}*{\mathrm{ml}}^{-1}$$	2500
Peripheral vascular resistance, Rper	$$\mathrm{Pa}*\mathrm{ms}*{\mathrm{ml}}^{-1}$$	80,000
Venous resistance, Rven	$$\mathrm{Pa}*\mathrm{ms}*{\mathrm{ml}}^{-1}$$	2000
Arterial compliance, Cart	$\mathrm{Pa}*\mathrm{ml}$	0.033
Venous compliance, Cven	$\mathrm{Pa}*\mathrm{ml}$	0.4
Resting volume for arterial, Vart,0	$\mathrm{ml}$	580
Resting volume for venous, Vven,0	$\mathrm{ml}$	3600
LA end-systolic elastance,${E}_{es,LA}$	$\mathrm{Pa}/\mathrm{ml}$	60
Volume axis intercept from ESPVR,${V}_{0,LA}$	$\mathrm{ml}$	10
Scaling factor for EDPVR,${A}_{LA}$	$\mathrm{Pa}$	58.67
Exponent coefficient for EDPVR,${B}_{LA}$	${\mathrm{ml}}^{-1}$	0.049
Time to LA end-systole,${t}_{max,LA}$	$\mathrm{ms}$	200
LA relaxation time constant,${\tau }_{LA}$	$\mathrm{ms}$	25

^5^).

### Section A3: Effects of shear stiffness ${B}_{{\varvec{f}}{\varvec{s}}}$ and ${B}_{{\varvec{f}}{\varvec{n}}}$ on LV functions and biomechanics

See Table 6

**Table 6:** Effects of shear stiffness ${B}_{{\varvec{f}}{\varvec{s}}}$ and ${B}_{{\varvec{f}}{\varvec{n}}}$ on LV functions and biomechanics for various FE simulations

TA 10$^\circ$		${B}_{{\varvec{f}}{\varvec{n}}}$ 19.2	${B}_{{\varvec{f}}{\varvec{n}}}$ 19.2	${B}_{{\varvec{f}}{\varvec{n}}}$ 14.4	${B}_{{\varvec{f}}{\varvec{n}}}$ 14.4	${B}_{{\varvec{f}}{\varvec{n}}}$ 19.2	${B}_{{\varvec{f}}{\varvec{n}}}$ 9.6	${B}_{{\varvec{f}}{\varvec{n}}}$ 9.6
${B}_{{\varvec{n}}{\varvec{s}}}$ 15.0		${B}_{{\varvec{f}}{\varvec{s}}}$ 19.2	${B}_{{\varvec{f}}{\varvec{s}}}$ 14.4	${B}_{{\varvec{f}}{\varvec{s}}}$ 19.2	${B}_{{\varvec{f}}{\varvec{s}}}$ 14.4	${B}_{{\varvec{f}}{\varvec{s}}}$ 9.6	${B}_{{\varvec{f}}{\varvec{s}}}$ 19.2	${B}_{{\varvec{f}}{\varvec{s}}}$ 9.6
Healthy (SA 18$^\circ$)	EF (%)	53.8	53.9	53.8	53.9	54.0	53.8	54.0
	SV (ml)	86.3	86.8	86.6	87.0	87.5	86.9	88.0
	Peak Systolic pressure	119.7	120.2	119.7	120.2	121.0	119.8	121.0
	Maximal Fiber Stress	55.1	54.9	55.4	55.2	54.9	56.0	55.7
HCM (SA 48$^\circ$)	EF	60.6	60.7	60.7	60.8	60.9	60.9	60.9
	SV	92.4	92.9	92.8	93.3	93.7	93.4	93.7
	Peak Systolic pressure	140.7	140.6	140.9	140.6	140.5	141.2	140.5
	Maximal Fiber Stress	32.1	31.7	32.5	32.2	31.2	33.3	31.2
DCM (SA 18$^\circ$)	EF	43.2	43.2	43.0	43.0	43.2	42.9	42.9
	SV	113.0	113.2	112.9	113.0	113.4	112.6	113.0
	Peak Systolic pressure	135.9	136.0	135.5	135.6	136.2	134.9	135.1
	Maximal Fiber Stress	77.7	77.6	77.8	77.7	77.6	78.2	77.9

*SA* sheetlet angle, *TA* transverse angle. Unit for peak systolic pressure is mmHg, and unit for maximal fiber stress is kPa

.

#### Effects of transverse angle on LV functions and biomechanics

See Fig. 10.

### Influences of contractility and stiffness on sheetlet sliding in HCM and DCM LV

See Figs. 11 , 12, and Table 7.

**Table 7:** Gradients of the relationships between shear stiffness ${B}_{{\varvec{n}}{\varvec{s}}}$ and various LV functions at different active tension and global stiffness values in HCM (Fig. 11) and DCM (Fig. 12)

LV geometry	Active tension and global stiffness	Gradient			
		EF (%)	SV (ml)	Peak systolic pressure (mmHg)	Maximal fiber stress (kPa)
HCM	T0 150 kPa, C 100 Pa	− 0.572	− 1.508	− 1.526	− 0.556
HCM	T0 105 kPa, C 100 Pa	− 0.522	− 1.446	− 1.342	− 0.526
HCM	T0 150 kPa, C 200 Pa	− 0.682	− 1.520	− 1.688	− 0.536
HCM	T0 105 kPa, C 200 Pa	− 0.646	− 1.462	− 1.460	− 0.508
HCM	T0 75 kPa, C 300 Pa	− 0.644	− 1.306	− 1.284	− 0.458
DCM	T0 150 kPa, C 100 Pa	− 0.020	− 0.198	− 0.154	− 0.190
DCM	T0 105 kPa, C 100 Pa	0.012	− 0.166	− 0.108	− 0.234
DCM	T0 150 kPa, C 200 Pa	− 0.060	− 0.334	− 0.266	− 0.282
DCM	T0 105 kPa, C 200 Pa	− 0.016	− 0.248	− 0.180	− 0.296

### Section A5: ${{\varvec{E}}}_{{\varvec{n}}{\varvec{s}}}$ and ${{\varvec{E}}}_{{\varvec{r}}{\varvec{r}}}$ shear strains during sheetlet sliding

To validate that sheetlet sliding was indeed the mechanism for the increased cardiac function when shear stiffness, ${B}_{{\varvec{n}}{\varvec{s}}}$, was decreased, we extracted Green strain components, ${{\varvec{E}}}_{{\varvec{n}}{\varvec{s}}}$ and ${{\varvec{E}}}_{{\varvec{r}}{\varvec{r}}}$, at a mid-wall, mid-ventricle location in the healthy LV simulation case, to verify that increased ${{\varvec{E}}}_{{\varvec{n}}{\varvec{s}}}$ strain (or sheetlet sliding strain) in fact occurred in cases with decreased ${B}_{{\varvec{n}}{\varvec{s}}}$ and increased cardiac function, and that this situation was also associated with increased ${{\varvec{E}}}_{{\varvec{r}}{\varvec{r}}}$. Results in Table

**Table 8:** Strain components at a mid-wall, mid-ventricle location in various healthy LV simulations

${B}_{{\varvec{n}}{\varvec{s}}}$	SA	max ${{\varvec{E}}}_{{\varvec{r}}{\varvec{r}}}$	max ${{\varvec{E}}}_{{\varvec{n}}{\varvec{s}}}$
9.3	0^o^	0.38	0.01
9.3	18^o^	0.40	0.23
9.3	48^o^	0.42	0.31
17.8	0^o^	0.38	0.01
17.8	18^o^	0.38	0.15
17.8	48^o^	0.37	0.27

^8^ showed that when SA = 0°, there was no difference in these strain components. However, when SA > 0°, a reduced ${B}_{{\varvec{n}}{\varvec{s}}}$ produced greater ${{\varvec{E}}}_{{\varvec{n}}{\varvec{s}}}$ and greater ${{\varvec{E}}}_{{\varvec{r}}{\varvec{r}}}$.

### Section A6: Analysis of myocardial sheetlet angle dynamics

From the FEM results, the sheetlet angle mobility in healthy LV was near 11$^\circ$, which was only about 25% of the measurements from (Nielles-Vallespin et al. 2017). We performed an idealized mathematical analysis to estimate the effects of myocardial deformation on sheetlet angle mobility, to understand how much sheetlet mobility could be accounted for by macroscopic deformation. The sheetlet angle dynamics was calculated using Eqs. 10a and 10b which described the sheetlet motion from diastolic sheet direction ***S*** to the systolic sheet direction ***S’*** (illustrated by Fig. 13). These equations assumed that sheetlet mobility is solely caused by macroscopic deformation, and there was no microstructural reorganization to change sheetlet orientations further.

10a $$\begin{aligned} {\varvec{S}} & = \alpha {\varvec{L}} + \beta {\varvec{C}} + \gamma {\varvec{R}} \\ {{\varvec S}^{\prime}} & = \lambda_{{\varvec{L}}} \alpha {\varvec{L}} + \lambda_{{\varvec{C}}} \beta {\varvec{C}} + \lambda_{{\varvec{R}}} \gamma {\varvec{R}} \\ \end{aligned}$$

10b $$\begin{aligned} \cos \left( {{\text{SA}}_{{{\text{dia}}}} } \right) & = \frac{{\sqrt {\alpha^{2} + \beta^{2} } }}{{\sqrt {\alpha^{2} + \beta^{2} + \gamma^{2} } }} \\ \cos \left( {{\text{SA}}_{{{\text{sys}}}} } \right) & = \frac{{\sqrt {\left( {\lambda_{{\varvec{L}}} \alpha } \right)^{2} + \left( {\lambda_{{\varvec{C}}} *\beta } \right)^{2} } }}{{\sqrt {\left( {\lambda_{{\varvec{L}}} \alpha } \right)^{2} + \left( {\lambda_{{\varvec{C}}} \beta } \right)^{2} + \left( {\lambda_{{\varvec{R}}} \gamma } \right)^{2} } }} \\ \end{aligned}$$

where ${\varvec{L}}$, ${\varvec{C}}$ and ${\varvec{R}}$ were the unit vectors in the longitudinal, circumferential and radial directions, $\alpha$ and $\beta$ and $\gamma$ were the magnitudes of three components of ***S*** along ${\varvec{L}}$, ${\varvec{C}}$ and ${\varvec{R}}$ directions. The ${\lambda }_{{\varvec{L}}}$, ${\lambda }_{{\varvec{C}}}$ and ${\lambda }_{{\varvec{R}}}$ were the stretches in the ${\varvec{L}}$, ${\varvec{C}}$ and ${\varvec{R}}$ directions during systolic contractile deformations, ${\mathrm{SA}}_{\mathrm{dia}}$ was the diastolic sheetlet angle, while ${\mathrm{SA}}_{\mathrm{sys}}$ was the systolic sheetlet angle.

Assuming the configuration of the healthy LV, diastolic sheetlet angle to be 18$^\circ$, and longitudinal and circumferential strain of − 0.140 and − 0.203, systolic sheetlet angles was calculated to be 30$^\circ$, indicating sheetlet angle mobilities of 12 $^\circ$, which was closing to the FE results (mobility of 8° at a mid-wall mid-ventricle location). Using Eq. 10, we further computed that to achieve a sheetlet angle mobility of 45$^\circ$ via continuum deformation alone, as was measured by Nielles-Vallespin et al. (2017), a radial stretch of 333% was needed, which was much larger than the 64% peak radial strain reported by Nielles-Vallespin et al. (2017).

### Section A7: Changes to maximum tensile stress components with changes to ${B}_{{\varvec{n}}{\varvec{s}}}$ stiffness

See Fig. 14.
